# Bottom‐up and top‐down effects combine to drive predator–prey interactions in a forest biodiversity experiment

**DOI:** 10.1111/1365-2656.70103

**Published:** 2025-07-07

**Authors:** Jing‐Ting Chen, Ming‐Qiang Wang, Arong Luo, Feng Zhang, Douglas Chesters, Shanlin Liu, Yi Li, Goddert von Oheimb, Matthias Kunz, Qing‐Song Zhou, Helge Bruelheide, Xiao‐Juan Liu, Ke‐Ping Ma, Andreas Schuldt, Chao‐Dong Zhu

**Affiliations:** ^1^ CAS State Key Laboratory of Animal Biodiversity Conservation and Integrated Pest Management (SKLA2501), Institute of Zoology, Chinese Academy of Sciences Beijing China; ^2^ College of Biological Sciences, University of Chinese Academy of Sciences Beijing China; ^3^ Forest Nature Conservation University of Göttingen Göttingen Germany; ^4^ Key Laboratory of Mountain Ecological Restoration and Bioresource Utilization & Ecological Restoration Biodiversity Conservation Key Laboratory of Sichuan Province Chengdu Institute of Biology, Chinese Academy of Sciences Chengdu China; ^5^ Department of Entomology College of Plant Protection, Nanjing Agricultural University Nanjing China; ^6^ State Key Laboratory of Vegetation and Environmental Change, Institute of Botany, Chinese Academy of Sciences Beijing China; ^7^ Technische Universität Dresden Institute of General Ecology and Environmental Protection Tharandt Germany; ^8^ Institute of Biology/Geobotany and Botanical Garden Martin Luther University Halle‐Wittenberg Halle (Saale) Germany; ^9^ German Centre for Integrative Biodiversity Research (iDiv) Halle‐Jena‐Leipzig Leipzig Germany; ^10^ State Key Laboratory of Integrated Pest Management Institute of Zoology, Chinese Academy of Sciences Beijing China

**Keywords:** arthropod, bottom‐up and top‐down control, diet, metabarcoding, predator–prey interaction, tree diversity, trophic interaction

## Abstract

1. The bottom‐up effect of producers and the top‐down effect of predators are well‐known factors shaping community assembly and ecosystem functioning through trophic interactions. Communities differing in their functional composition may induce ecological effects with varying directions and intensities, but previous studies in highly diverse ecosystems have struggled with reliably quantifying these interactions at the community level.
2. We used spider gut‐content metabarcoding in a subtropical tree diversity experiment to examine the impact of multiple diversity components of both trees and spiders on prey diversity and the network structure of predator–prey interactions.
3. Our findings reveal that prey richness and spider‐prey network structure are simultaneously driven by the bottom‐up effects of tree communities and the top‐down effects of the spider communities. When categorized by hunting modes, the drivers of prey richness and network structure differed between spider guilds. Large phylogenetic and functional differences within web‐building spider communities promoted coexistence, leading to increases in the utilized prey richness, generality, niche overlap and prey vulnerability. For hunting spiders, the effects of vertical tree structure complexity indicated restricted mobility but facilitated coexistence through increased shelter availability, and a concomitant reduction of prey richness and dietary breadth.
4. Our study underscores the significance of integrating multiple diversity components and considering functional trait composition across trophic levels when analysing the ecological effects of generalist predators. Our findings enable a better understanding of how predator–prey interaction patterns may be altered under current environmental changes that result in biodiversity loss.

## Graphical abstract

This study constructed a real spider–prey interaction network using metabarcoding, integrating multitrophic levels (Plant–Prey–Spider) and multiple diversity indices (taxonomic, functional and phylogenetic diversity) to analyse how bottom‐up effects from tree communities and top‐down effects from spider communities influence spider–prey interactions in a subtropical forest.

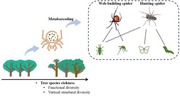

## 1 INTRODUCTION

The interactions between predators and their prey constitute crucial links connecting community dynamics across trophic levels (Finke & Denno, 2004; Schmitz, 2007). In this process, the effects of predator diversity and community composition cascade down from prey to primary producers, ultimately regulating ecosystem functions such as productivity (Miller et al., 2006; Schmitz, 2009), pest control (Snyder et al., 2006) and decomposition (Hawlena et al., 2012). At the same time, the structure and diversity of predator communities, and thus the strength of top‐down control, can depend on the bottom‐up effects of their prey communities and the plants providing basal ecosystem resources (Leles et al., 2017). Human alterations of ecosystems and climate change are transforming these communities and causing biodiversity loss (Johnson et al., 2017; Kardol et al., 2018), but understanding how this affects interactions across trophic levels remains challenging for the highly diverse communities of arthropods in many ecosystems (Eisenhauer et al., 2019; Roslin et al., 2017).

Previous research has highlighted how plant diversity loss cascades through food webs, significantly reducing arthropod diversity across trophic levels and altering arthropod community structure (Haddad et al., 2009; Scherber et al., 2010). High plant diversity is expected to strongly influence the structure of plant–herbivore interaction networks, including connectance, generality and long‐term stability (Welti et al., 2017). While plant and herbivore diversity are often correlated with predator diversity and shape predator community composition (Root, 1973; Schuldt et al., 2019), structural changes in predator–prey networks are anticipated but remain poorly understood. For generalist predators, increased plant diversity may enhance prey diversity, leading to more frequent predator–prey interactions and potentially fostering apparent and exploitative competition, thereby subjecting prey to higher predation pressure from predators with broader diets (Giling et al., 2019; Otto et al., 2008). Studies suggest that plant diversity enhances herbivore movement and predation vulnerability (Straub et al., 2014). Quantifying changes in predator–prey network structure can elucidate the bottom‐up effects of plant communities on these networks.

There is still a dearth of studies examining changes in the interactions within real networks based on actual feeding relationships of arthropod predators and their prey (Giling et al., 2019). Due to the small body size, high motility and a diverse diet spectrum in arthropod predators, obtaining the prey spectrum using traditional methods (e.g. field observation and examination of tissue residue in gut and faeces; Sunderland, 1975) is challenging, particularly for fluid‐feeding arthropods such as spiders. Metabarcoding, however, offers a powerful alternative by enabling the quantification of prey diversity through high‐throughput sequencing of predator gut contents (De Barba et al., 2014; Galan et al., 2018). Well‐resolved networks offer the possibility to gain mechanistic insights into the effects of plant diversity on the structure and stability of multitrophic communities. Metabarcoding therefore provides opportunities to gain a more comprehensive understanding of the pathways and mechanisms of interactions between predators and other trophic levels (de Sousa et al., 2019; Galan et al., 2018).

Being able to quantify changes in predator–prey interactions with more confidence is also key to better understanding the role that predator diversity and species composition play in multitrophic interactions. This is important because bottom‐up effects of plant communities have been shown to become weaker at higher trophic levels (Scherber et al., 2010; Schuldt et al., 2019), and the Enemies Hypothesis remains a contested concept especially in forest ecosystems (Staab & Schuldt, 2020). In part, this may be because the nature and structure of predator–prey interactions can be altered due to variations in the functional traits of predators (Belgrad & Griffen, 2016; Brose et al., 2019). Prey selection by predators depends on their functional traits, such as body size (Osenberg & Mittelbach, 1989), mobility (Luttbeg et al., 2020) and gape limitation (Woodward & Hildrew, 2002), which ultimately determine predation success and intake of energy and nutrients. Additionally, predator hunting mode strongly determines the direction of trophic interactions (Luttbeg et al., 2020; Schmitz, 2009). Sit‐and‐wait predators, such as web‐building spiders, favour capturing actively mobile prey, while actively roaming predators, such as many active hunting spiders, favour sedentary prey (Ross & Winterhalder, 2015). It is thus important to consider the functional attributes of both predators and prey. Predators can enhance plant biomass and reduce herbivory and decomposition through consumptive and non‐consumptive effects (Preisser et al., 2005), whereas intraguild predation among predators may either slow down or reverse these effects (Sanders et al., 2011; Sitvarin & Rypstra, 2014).

While an increasing number of studies have demonstrated that trait‐based approaches, often using functional or phylogenetic diversity, offer advantages in elucidating interaction mechanisms at multiple trophic levels (Alavez et al., 2023; Bello et al., 2023), current analyses of interaction networks are predominantly confined to taxonomic diversity (Rzanny & Voigt, 2012; Scherber et al., 2010). Functional diversity mirrors the morphological and behavioural adaptations that species undergo to cope with environmental changes (Spasojevic & Suding, 2012). Phylogenetic diversity encompasses the evolutionary history and phylogenetic relationships of species (Singer et al., 2018; Webb et al., 2002) and serves as a functional diversity proxy when trait conservatism exists. The combination of a multitrophic perspective with interaction networks using trait‐based and phylogeny‐based approaches can be seen as a breakthrough for advancing biodiversity–ecosystem functioning research.

In this study, we examine the impact of tree diversity and predator diversity on prey diversity and predator–prey network structure in a large‐scale subtropical forest experiment (BEF‐China; Bruelheide, 2014), using a multifaceted approach that integrates taxonomic, functional and phylogenetic diversity with predator gut‐content metabarcoding. Spiders were chosen as the model predators due to their primary importance in many ecosystems (Michalko et al., 2019; Nyffeler & Birkhofer, 2017), representing the most abundant and diverse generalist predators with decisive effects on lower trophic levels at our study site (Chen et al., 2023). We used three metrics (generality, vulnerability and niche overlap) to evaluate the effect of tree diversity on the food web structure of predator–prey interactions. Generality measures the average number of prey species per predator, whereas vulnerability reflects the average number of predator species preying on a given prey species. Niche overlap quantifies the similarity in prey use among predator species. We hypothesized that (a) bottom‐up effects of tree communities provide diverse prey for spiders and promote more frequent spider–prey interactions at higher levels of tree diversity (i.e. a broader prey spectrum of spiders leading to increased prey sharing among spider species). As spiders are predators that do not feed on plants, we expected that, at the same time, (b) top‐down effects of spider diversity promote prey diversity captured by spiders and alter predator–prey network structure (i.e. niche width and overlap in prey use of spiders decrease). Additionally, (c) spiders with different hunting modes exhibit distinct mechanisms for regulating prey diversity and network structures. Specifically, prey richness and network structure of web‐building spiders (which rely on physical structures for web construction) are expected to be primarily driven by tree diversity, whereas those of hunting spiders (free‐wandering) are driven by spider community composition due to a higher likelihood of intraguild encounters. Finally, we expected these relationships to be (d) driven to a greater extent by functional and phylogenetic diversity measures than purely by taxonomic diversity.

## 2 MATERIALS AND METHODS

### 2.1 Study site and sampling

The study was conducted at the ‘BEF‐China’ experimental site (Bruelheide, 2014), located in Xingangshan, Jiangxi Province, China (29°08′–29°11′ N, 117°90′–117°93′ E). This research was conducted in a selection of plots representing random extinction scenarios according to a ‘broken‐stick’ design: based on a pool of 16 tree species, the set of 16 tree species was divided into two non‐overlapping sets, each consisting of 8 species, which subsequently were split again to yield plots with 16, 8, 4, 2 and 1 tree species. An additional tree species richness level, which comprised 24 species (eight additional species added to the 16‐species plots) was also included. In total, 55 plots (26 plots with 1 tree species, 13 plots with 2 tree species, 8 plots with 4 tree species, 4 plots with 8 tree species, 2 plots with 16 tree species and 2 plots with 24 tree species) were sampled, excluding plots with high tree mortality. Each plot is 25.8 × 25.8 m in size, with 400 trees planted in a grid of 20 × 20 trees with a 1.29 m distance between trees.

We collected spiders by beating branches of trees with a stick and using a sheet (1.5 × 1.5 m) to collect fallen arthropods. The collecting started from the first line per plot until we sampled 80 alive trees. We tried to standardize the volume of branches that were struck from each tree, employing a consistent method of striking each tree five times. Sampling was conducted on sunny days during the peak time of spider activity across all 55 plots in June 2017. All spiders were individually preserved in 99% ethanol at a temperature of −20°C in the laboratory for subsequent analysis. All spiders were identified using both morphological and molecular methods. The abdomens of all spiders were individually sequenced with metabarcoding to establish the real spider–prey interaction networks. This study exclusively involved invertebrate (Arachnid) specimens, which do not require special permits or animal care approvals for field collection or laboratory analysis.

### 2.2 Molecular workflow

All spiders were classified into morphotypes based on their morphological characteristics. To validate morphological classification, three individuals per group were randomly chosen to obtain DNA barcodes from their legs, with universal primers (LCO1490 and HCO2198) and Sanger sequencing. DNA extraction from spider legs followed the standard protocol of the TIANamp Genomic DNA Kit (TIANGEN, Beijing, China). The spider genomic DNA was amplified in 30 μL reaction volumes, containing 15 μL Premix PrimeSTAR HS (TaKaRa), 10 μL ddH2O, 1 μL forward/reverse primers and 3 μL template DNA. Thermocycling conditions were as follows: 94°C for 4 min, followed by 35 cycles at 94°C for 45 s, 45°C for 40 s, 72°C for 2 min and a final extension at 72°C for 10 min. All PCR products were visualized on a 1% agarose gel, and sequenced with BigDye v3.1 on an ABI 3730xl DNA Analyser (Applied Biosystems).

To construct a network of spider–prey interactions, each spider sample was washed with ethanol and individually weighed after drying (allowing it to stand in an isolated sterile environment at room temperature for 2 h). Then the abdomens of the spiders were ground thoroughly with a grinding rod after being frozen by liquid nitrogen and subjected to DNA extraction following the standard experimental process of QIGEN DNeasy Blood & Tissue Kit (ID: 69504). As prey DNA in spider guts is highly degradable and fragmented, ZBJ‐ArtF1c and ZBJ‐ArtR2c primers were used, targeting a minibarcode of 157 bp in the mitochondrial COI gene (Zeale et al., 2011). The 5′ ends of both forward and reverse primers were ligated to unique barcode sequences (Table S6). The barcodes were eight nucleotides in length and there were at least four nucleotide mismatches between barcodes. Each PCR amplification was matched with a pair of certain barcodes (e.g. F1‐R1, F2‐R2) to ensure the sequences were assigned to the corresponding sample in sebsequent analysis. Forty samples, including a negative control and three replicates each, were processed in a PCR round to ensure that only successfully amplified PCR products were used for subsequent experiments. Each PCR tube contained 18 μL Premix PrimeSTAR HS (TaKaRa), 3 μL ddH_2_O, 1 μL forward/reverse primers and 2 μL template DNA. PCR conditions were as follows: 95°C for 3 min, followed by 30 cycles at 94°C for 30 s, 50°C for 30 s, 65°C for 2 min and a final extension at 65°C for 5 min. PCR products were visualized by electrophoresis on 1% agarose gels and then purified with a PCR purified kit (Vazyme). The purified PCR products were quantified using a Qubit Fluorometer. The amplicons for each sample were mixed according to the next high‐throughput sequencing requirements and finally sequenced in an Illumina Novaseq 6000 high‐throughput instrument (2 × 150 bp paired‐end reads).

### 2.3 Bioinformatics analysis

Sequences extracted from the spider legs were aligned with MAFFT v 7.0 (Katoh et al., 2002), edited with BioEdit v 7.0.5 and checked for the presence of stop codons with MEGA X (Kumar et al., 2018). Five methods (ABGD, BLASTclust, jMOTU, Mothur, PTP) were used to delimit Molecular Operational Taxonomic Units (MOTU, Blaxter et al., 2005). The Hubert and Arabie‐adjusted Rand index was computed using the R package clues (Wang et al., 2007) to identify the most consistent species delimitation approach. We developed a custom spider reference database by integrating the morphological taxonomy and COI sequence per adult specimen. All morphological identifications were conducted by Professor Jie Liu (a spider taxonomic expert) at Hubei University. The immature spiders were identified based on the local and BOLD (The Barcode of Life Data System; Ratnasingham & Hebert, 2007) online reference data, with Statistical Assignment Package (SAP; Munch, Boomsma, Huelsenbeck, et al., 2008; Munch, Boomsma, Willerslev, et al., 2008).

Raw reads of metabarcoding were initially quality‐checked using FastQC v0.11.9 (Andrews, 2015). Forward and reverse reads were then merged using VSEARCH v 2.22.1 (Rognes et al., 2016), with a minimum overlap of 73 bp base pairs. Sequences were demultiplexed into their respective samples based on sample‐specific barcodes, applying a zero‐mismatch threshold. Primer and barcode sequences were trimmed using CUTADAPT v4.2 (Martin, 2011). Subsequently, sequences from each sample were length‐filtered to retain reads of 157 bp, dereplicated and clustered at a 97% identity threshold. As the entire abdomen of spiders was used for DNA extraction, a significant proportion of predator (spider) reads were present in the sequencing data. To remove these host‐derived reads, we utilized BLAST 2.12.0+ (basic local alignment search tool, Sayers et al., 2022) with a 95% identity threshold against a reference database. This database comprised sequences obtained from spider legs and the most abundant sequence from each sample in the metabarcoding analysis. To remove the potential cross‐contamination during molecular experiments, we performed a secondary sequence filtration using host spider sequences from one experimental group (40 samples) as the reference database. Then, reads from all samples were pooled and dereplicated to generate a catalogue of unique putative haplotypes. Denoizing was performed using UNOISE3, with numbers of reads fewer than five removed. Chimeras were filtered de novo using the UCHIME3 algorithm built‐in VESEARCH. Sequences were clustered into MOTUs at 97% and taxonomically assigned using SAP through a local arthropod database downloaded from the BOLD system. Taxonomic assignments were further validated using the NCBI nr/nt database. The closest matching hit with the lowest E‐value was assigned as the final taxonomic identification for each MOTU (Cuff et al., 2021). Finally, we normalized the prey OTUs across samples and removed OTUs with fewer than eight reads and a relative abundance lower than 0.01.

### 2.4 Spider phylogeny and functional traits

We constructed the phylogenetic tree of spiders using COI sequences derived from spider legs. To enhance the structure of the phylogeny, we incorporated previously published topologies (Fernández et al., 2018; Garrison et al., 2016; Kallal et al., 2021) to constrain the trunk and terminal branches of the phylogenetic tree, following the methodological framework outlined by Chesters (2020). The predation efficiency and survivability of spiders depend on morphological and behavioural characteristics. Therefore, we measured and calculated three morphological traits of five randomly chosen individuals per species, including body shape (body length divided by width of carapace), flatness (height of carapace divided by body length) and biomass (mean dry weight), which are linked to physical activity (locomotion, dispersal, space use and thermoregulation; Ferreira‐Sousa et al., 2021; Jenkins et al., 2007; Jetz et al., 2004) and resource preferences (Birkhofer et al., 2022) (Table S1). Additionally, we categorized phenology into three levels, based on the occurrence of spider MOTUs sampled across three seasons (April, June and September 2017), indicating the duration that spiders exert hunting pressure on their prey (Urban, 2007). Spider sampling and identification across all three seasons followed identical experimental protocols (Chen et al., 2023). We also included the hunting mode of spiders (web‐building or active hunting), which is an important indicator reflecting different interaction pathways between spiders and other trophic levels (Schmitz, 2003; Schmitz, 2008).

### 2.5 Statistical analyses

All analyses were performed with software R 4.3.2 (www.r‐project.org) with the packages bipartite (Dormann et al., 2008), FD (Laliberté & Legendre, 2010) and picante (Kembel et al., 2010). The analyses were conducted using data aggregated at the plot level. All analyses were performed on three datasets (all, web‐building and hunting spiders), as spiders in different hunting modes have different functions and prey search strategies (Michalko & Pekár, 2016).

A variety of plant traits that have been shown to influence the diversity and community structure of arthropods (both of predators or their prey) by determining nutritional conditions and habitat structure were included in our analysis (Langellotto & Denno, 2004; Schuldt et al., 2019) (Table S2). The plant vertical structural diversity (VD) was included in our analysis as an indicator of the structural heterogeneity of the plant community at the plot level, which has been proven to influence spider community assembly, especially web‐building spiders (Ávila et al., 2017; Greenstone, 1984). We quantified phylogenetic diversity using phylogenetic mean pairwise distance (MPD, mean evolutionary distance among all pairs of species) with R package picante (Webb et al., 2002). We used Rao's quadratic entropy to quantify the functional diversity of trees and spiders using the FD package with Gower distance. We weighted the phylogenetic and functional values of trees by tree volume, while weighting spiders' phylogenetic and functional values by abundance (Laliberté & Legendre, 2010). Tree volume was determined by utilizing the basal area and height measurements of the central subplot, collected in October 2016 (Fichtner et al., 2017). More details on plant diversity are given in Supporting Information.

Quantitative spider‐prey food webs were constructed based on MOTUs of spiders and prey using the bipartite package (Dormann et al., 2008). Generality, vulnerability and niche overlap were used to evaluate the structure and stability of the networks. To ensure robust estimates, network indices were calculated only for plots with at least three spider species with detected prey content. We then applied Patefield null models (10,000 simulations) to compare observed indices with random expectations (Dormann et al., 2009), testing whether network indices significantly deviated from chance across all plots.

We used linear regression models to examine the relationships of each of the response variables (prey richness, generality, vulnerability, niche overlap) with tree and spider diversity. Models were run separately for data on all spiders and for data on spiders with either web‐building or active hunting mode. Predictors included species richness, functional diversity (FD) and vertical structural diversity (VD) of the tree communities, as well as abundance and mean phylogenetic distance (MPD) of corresponding spider communities in the models. Spider abundance was included as a predictor variable in all models to account for potential differences in sampling intensity. We also considered study site (site A and site B) as a predictor to account for site‐specific differences in tree and spider community composition. To avoid potential multicollinearity, we checked correlations among predictors and calculated variance inflation factors (VIF < 3). Due to a strong correlation between phylogenetic MPD and functional diversity of spiders (All: MPD vs. FD = 0.71, Web‐building: MPD vs. FD = 0.71, Hunting: MPD vs. FD = 0.46; Figure S1), only phylogenetic MPD was retained in the models. Model residuals were checked for normality and homoscedasticity. To satisfy linearity assumptions, spider abundance, generality (all spiders) and vulnerability were log‐transformed, while the remaining response variables were square‐root transformed. Initial regression models were simplified through a stepwise process based on AICc values until the best‐fitting model with the lowest AICc value was obtained.

## 3 RESULTS

In total, we collected 1540 spiders, comprising 115 spider OTUs. The raw sequencing data from metabarcoding comprised 648,353,960 reads, with 313,137,146 reads remaining after merging. Among them, the guts of 512 spiders contained at least one prey species from a total of 442 detected prey OTUs. The prey groups mainly included Araneae, Diptera, Lepidoptera, Hemiptera, Blattodea, Collembola, Orthoptera.

For null model analysis, observed network metrics significantly deviated from random expectations on all or most of the study plots, indicating that species interactions were not driven by random processes (Tables S3–S5).

**FIGURE 1:**
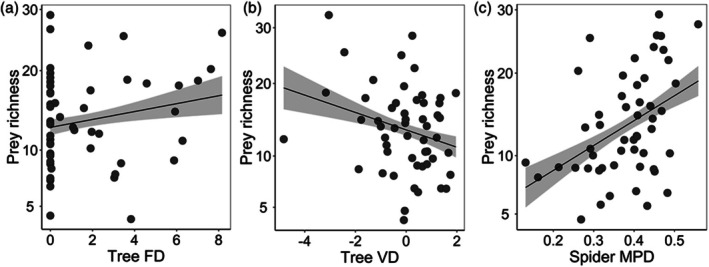
Relationships between prey richness and (a) tree functional diversity (FD), (b) tree vertical structural diversity (VD) and (c) mean pairwise phylogenetic distance (MPD) of all spiders. The regression lines indicate significant relationships at *p* < 0.05 (with 95% confidence bands).

**FIGURE 2:**
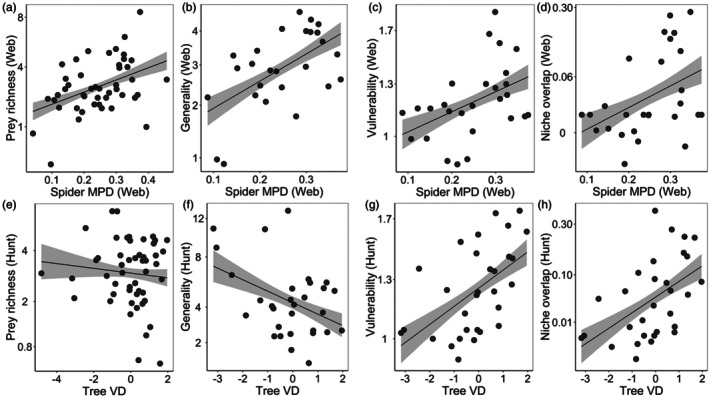
Relationships of (a, e) prey richness, (b, f) generality, (c, g) vulnerability, (d, h) niche overlap with the specific key explanatory variables for web‐building spiders (a–d) and actively hunting spiders (e–h). Web‐building spiders display strong relationships to spider phylogenetic diversity (MPD), while active hunting spiders more respond to tree vertical structural diversity (VD). The regression lines indicate significant relationships at *p* < 0.05 (with 95% confidence bands).

For all spiders, prey richness, vulnerability and niche overlap showed a consistently significant relationship with tree FD. Tree VD was negatively related to prey richness but positively related to vulnerability (Figure 1; Table 1). Spider phylogenetic MPD was positively related to prey richness and vulnerability and showed a near‐significant positive relationship with niche overlap (Table 1). For web‐building spiders, spider phylogenetic MPD showed a positive and significant relationship with prey richness and network indices (generality, vulnerability and niche overlap; Figure 2a–d; Table 1). Additionally, tree FD was positively related to vulnerability and niche overlap (Table 1). For active hunting spiders, tree VD exhibited a negative and significant relationship with prey richness and generality (Figure 2e–f; Table 1) but a positive relationship with vulnerability and niche overlap (Figure 2g–h; Table 1). Tree richness was negatively related to prey richness of active hunting spiders (Table 1). Furthermore, hunting spider phylogenetic MPD was negatively and significantly associated with generality of spider–prey interactions (Table 1). Spider abundance consistently exhibited a positive and significant correlation with prey diversity and multiple network indices of all spiders (Table 1). Additionally, it showed a positive correlation with prey richness and generality for both active hunting and web‐building spiders (Table 1).

**TABLE 1:** Summary results of linear models for prey richness, generality, vulnerability and niche overlap of spider communities (all, web‐building and hunting) in relation to tree diversity (species richness, functional diversity and vertical structural diversity) and spider diversity (abundance and phylogenetic diversity).

	All				Web‐building				Hunting			
	Est	SE	*t*	*p*	Est	SE	*t*	*p*	Est	SE	*t*	*p*
Prey richness												
(Intercept)	3.64	0.11	33.80	<0.001	2.52	0.11	22.52	<0.001	2.87	0.12	24.50	<0.001
Tree richness	−0.29	0.16	−1.79	0.081	—	—	—	—	−0.42	0.18	−2.27	<0.05
Tree FD	0.35	0.16	2.14	<0.05	—	—	—	—	0.38	0.19	2.00	0.052
Tree VD	−0.24	0.12	−2.08	<0.05	—	—	—	—	−0.30	0.13	−2.40	<0.05
Spider abundance	0.72	0.12	6.07	<0.001	—	—	—	—	—	—	—	—
Spider abundance (Web)	—	—	—	—	0.58	0.11	5.16	<0.001	—	—	—	—
Spider abundance (Hunt)	—	—	—	—	—	—	—	—	0.66	0.14	4.90	<0.001
Spider MPD	0.38	0.11	3.34	<0.01	—	—	—	—	—	—	—	—
Spider MPD (Web)	—	—	—	—	0.31	0.11	2.76	<0.01	—	—	—	—
Generality												
(Intercept)	1.35	0.08	17.06	<0.001	1.71	0.05	34.22	<0.001	2.10	0.11	19.93	<0.001
Tree VD	—	—	—	—	—	—	—	—	−0.25	0.11	−2.34	<0.05
Spider abundance (Web)	—	—	—	—	0.10	0.05	2.04	0.053	—	—	—	—
Spider abundance (Hunt)	—	—	—	—	—	—	—	—	0.25	0.12	2.13	<0.05
Spider MPD (Web)	—	—	—	—	0.18	0.05	3.48	<0.01	—	—	—	—
Spider MPD (Hunt)	—	—	—	—	—	—	—	—	−0.27	0.12	−2.30	<0.05
Vulnerability												
(Intercept)	0.35	0.04	9.07	<0.001	0.23	0.05	5.11	<0.001	0.21	0.03	6.21	<0.001
Tree FD	0.07	0.03	2.47	<0.05	0.08	0.04	2.10	<0.05	—	—	—	—
Tree VD	0.08	0.03	2.64	<0.05	0.08	0.04	2.06	0.051	0.10	0.04	2.90	<0.01
Site B	−0.11	0.07	−1.64	0.110	−0.17	0.08	−2.07	0.050	—	—	—	—
Spider abundance	0.11	0.03	3.70	<0.001	—	—	—	—	—	—	—	—
Spider MPD	0.08	0.03	2.68	<0.05	—	—	—	—	—	—	—	—
Spider MPD (Web)	—	—	—	—	0.09	0.04	2.42	<0.05	—	—	—	—
Spider MPD (Hunt)	—	—	—	—	—	—	—	—	0.06	0.04	1.77	0.087
Niche overlap												
(Intercept)	0.20	0.02	9.83	<0.001	0.16	0.03	4.64	<0.001	0.21	0.03	6.18	<0.001
Tree FD	0.05	0.02	2.28	<0.05	0.11	0.03	3.16	<0.01	—	—	—	—
Tree VD					—	—	—	—	0.09	0.03	2.63	<0.05
Spider MPD	0.03	0.02	1.70	0.096	—	—	—	—	—	—	—	—
Spider MPD (Web)	—	—	—	—	0.08	0.03	2.28	<0.05	—	—	—	—

*Note*: The table shows standardized parameter estimates (with standard error, *t* and *p* values) for variables retained in the minimal model after backward selection. Predictors marked with ‘—’ were excluded from the final model.

## 4 DISCUSSION

Our study elucidates the effects of tree and spider diversity on the prey diversity and network structure of spider–prey interactions. The results suggest that both bottom‐up effects of tree communities and top‐down effects of spider communities impact prey diversity and network interactions across all spider groups. However, when spiders were partitioned by hunting mode, the primary influencing factors differed, suggesting that different mechanisms underlie the potential for top‐down control by spider guilds. The prey richness and network structures of web‐building spiders were primarily driven by their community phylogenetic diversity, whereas those of hunting spiders were most influenced by the vertical stratification of tree communities. Our results thus emphasize the importance of addressing both bottom‐up and top‐down processes across multiple trophic levels by incorporating functional groups, multiple diversity components and modern molecular methods.

Our findings highlight that spider–prey interactions are shaped by the diversity of both tree and spider communities, with the direction of these effects being primarily driven by phylogenetic, functional and vertical structural diversity. Tree functional diversity constantly enhanced prey richness for all spiders while also increasing prey sharing and niche overlap among them. Two possible mechanisms might be at play here, related to the availability of prey and niche space, respectively, conforming with the predictions of the Enemies hypothesis (Root, 1973) that heterogeneous environments offer increased food resources and habitat for predators. Regarding the first mechanism, functionally and structurally more diverse tree communities offer abundant prey resources for predatory spiders (Schuldt et al., 2014; Wang et al., 2019; Xiao et al., 2020). Diverse prey communities can meet the dietary requirements of a more diverse spider community (Greenop et al., 2018). With respect to the second mechanism, tree diversity can promote the diversity of available habitats and shelter, allowing for a more diverse predator community to coexist and target similar prey (Chen et al., 2023; Schuldt et al., 2019). However, the vertical structural complexity of tree communities reduces prey richness captured by spiders, likely due to environmental complexity constraining their predation efficiency.

Spider communities with distant phylogenetic relationships captured more diverse prey, with more spider species sharing similar prey resources (high vulnerability). Distant phylogenetic relationships typically reflect relatively higher evolutionary divergence, implying that co‐occurring species exhibit enhanced functional divergence and niche differentiation (Srivastava et al., 2012). This differentiation arises in traits like habitat preferences, activity timing or predation strategies (Cardoso et al., 2011; Gonçalves‐Souza et al., 2014), thereby enhancing the prey richness of total spider communities. Studies have revealed that different spider species exhibit distinct preferences and avoidances in prey selection (Mezőfi et al., 2020). The stronger niche overlap of prey resources of distantly related spiders in our study may therefore seem unexpected. However, spiders exhibit a highly redundant prey spectrum across species, although distinct prey selection preferences exist among different species (Cardoso et al., 2011; Roubinet et al., 2018). Consequently, when spiders with distant phylogenetic relationships coexist, their distinct prey preferences distribute predation pressure across shared prey, promoting species coexistence of spiders and increasing overall predation pressure on prey communities.

Hunting mode, a crucial functional trait in spiders, regulates lower trophic levels through various pathways (Schmitz, 2003, 2008), as also clearly demonstrated in our study. Phylogenetically and functionally diverse web‐building spider communities captured a wider range of prey, with a wider prey spectrum per spider species (higher generality) and showed higher prey sharing and similarity among species (higher vulnerability and niche overlap). Web‐building spiders, characterized by their limited mobility, primarily rely on constructing webs to wait for prey, heavily influenced by environmental structure and food availability (Mcnett & Rypstra, 2000). Spider webs of varying structures and sizes occupy distinct ecological niches within the ecosystem, a feature that shows strong phylogenetic conservatism in web‐building spider families (Cardoso et al., 2011). Therefore, functionally and phylogenetically diverse communities of web‐building spiders enhance space utilization, increasing encounters with a wider array of prey (Bonte et al., 2008; Fasola & Mogavero, 1995). Functionally diverse tree communities (high tree FD) increased prey similarity among web‐building spiders (high niche overlap), thereby elevating predation pressure on prey (high vulnerability). Structurally complex and diverse habitats may provide niches for web‐building spiders, reducing interspecific competition and facilitating coexistence. Our results of positive effects of tree functional diversity and spider phylogenetic diversity thus indicate that the bottom‐up effects of tree diversity are reinforced by a simultaneous increase in top‐down effects by the web‐spider communities.

In contrast to web‐builders and to our expectations, the phylogenetic diversity of actively hunting spiders was less related to prey richness and predator–prey interactions, while the effect of tree vertical stratification was more pronounced. Hunting spiders rely on nimble mobility and rapid pounces to capture prey (Bonte et al., 2008; Fasola & Mogavero, 1995; Miller et al., 2014; Wise, 2006). Their predation success is thus primarily driven by locomotor performance and foraging tactics, which are less related to phylogeny (Mezőfi et al., 2020). This might be the reason why hunting spider phylogenetic diversity was largely unrelated to prey richness and network indices. Rather, these metrics were strongly linked to tree vertical structural diversity. Increasing vertical complexity of tree communities decreased the prey richness and average prey spectrum (generality) of hunting spiders, while it increased the overall similarity of prey spectra across different species (vulnerability and nice overlap). Structural complexity may act as a double‐edged sword for predators, constraining the mobility of hunting spiders and dispersal of insects, while at the same time providing more opportunities for retreat and hiding places that reduce predator encounter (da Silva Filho et al., 2024). Structurally complex environments offer varied refuge for prey (van Schalkwyk et al., 2021). This complexity may prolong search time and heighten the difficulty for hunting spiders to locate prey, thereby diminishing their predation efficiency. Prey that seek refuge are less likely to be located and captured than prey that frequently move (Sommer & Schmitz, 2020). Furthermore, hunting spiders are highly mobile and exhibit significant levels of intraguild predation (Petráková et al., 2016). Vegetation structural complexity reduces the probability of encounters among hunting spiders, which, by avoiding competition and intraguild predation (Finke & Denno, 2002), may facilitate their coexistence. Proximity in physical space enables different hunting spider species to capture similar prey more easily, leading to greater niche overlap in their diets. Simultaneously, we observed a decline in prey richness for hunting spiders with increasing tree species richness. The effects of tree diversity besides the effect of increased complexity (tree VD) are less clear because tree richness and functional diversity effects counteract each other, indicating that further research into the mechanisms behind tree diversity effects is required. The only effect of hunting spider phylogenetic MPD was a reduction in the generality of spider–prey interactions due to increased phylogenetic diversity within the spider communities. That is, the co‐occurrence of phylogenetically distant hunting spiders decreased the average number of prey species captured per spider species. Although spiders with similar phylogenetic relationships are often assumed to utilize similar resources, studies have demonstrated that prey composition in hunting spiders varies significantly among closely related species, while greater similarity is observed among phylogenetically distant species (Mezőfi et al., 2020). These contrasting results for web‐building and hunting spiders highlight the key role of the predator hunting mode in determining the interplay between bottom‐up and top‐down effects.

Finally, our findings show that spider abundance consistently and significantly influenced both prey richness and network structure. Spider abundance in our models was primarily used to control for differences in sample size across study plots. Nevertheless, the results underscore that increased predator abundance leads to stronger top‐down predation pressure, which is highly relevant when considering that abundances and biomass have been reported to particularly strongly respond to human‐induced ecosystem alterations in ecosystems (Staab et al., 2023).

## 5 CONCLUSIONS

Our study shows that the top‐down effects of spider communities on their prey are simultaneously driven by tree diversity and spider composition. However, the primary drivers vary among spiders with different hunting modes: spider phylogenetic diversity is the main driver for web‐builders, whereas tree vertical stratification is the most significant driver for hunting spiders. These findings underscore the significant role of multitrophic interactions between plants, predators and their prey—in particular with respect to their functional and phylogenetic composition—in collectively regulating relationships between biodiversity and ecosystem functions through bottom‐up and top‐down effects. Molecular gut‐content analyses allow for the reconstruction of real predator–prey interaction networks, enabling a deeper understanding of highly diverse ecosystems. Our research advocates for a closer consideration of community composition according to the biological traits of generalist predators, such as spiders, when evaluating their role in ecosystem stability and productivity enhancement. Prey serve as crucial mediators of predators' top‐down effects on plant communities, thereby regulating ecosystem functions. The diversity, biomass and composition of potential prey communities directly influence prey selection and interaction pathways of predators (Preston et al., 2019; Shultz et al., 2004), especially generalists. While we constructed the real spider–prey interactions with metabarcoding in this study, it did not account for environmental prey community characteristics, a limitation that needs to be addressed through expanded sampling in future research.

## AUTHOR CONTRIBUTIONS

Andreas Schuldt, Chao‐Dong Zhu and Jing‐Ting Chen conceived the idea; Chao‐Dong Zhu, Andreas Schuldt and Jing‐Ting Chen designed the research; all authors collected and/or contributed data and advice. Jing‐Ting Chen, Feng Zhang and Shan‐Lin Liu conducted the molecular experiment and bioinformatics analysis of high‐throughput sequences; Jing‐Ting Chen and Douglas Chesters conducted the spider phylogeny construction; Goddert von Oheimb and Matthias Kunz contributed the data on tree structure diversity; Jing‐Ting Chen and Ming‐Qiang Wang conducted the statistical analyses; Jing‐Ting Chen wrote the manuscript with input from Andreas Schuldt and other authors.

## CONFLICT OF INTEREST STATEMENT

The authors declare no conflicts of interest.

## Supporting information


**Table S1:** The functional traits, measurement and ecological functions of spiders.
**Table S2:** The functional traits and ecological functions of leaf traits.
**Table S3:** The comparison between observed network indices (generality, vulnerability, and niche overlap) and simulated network indices of all spiders.
**Table S4:** The comparison between observed network indices (generality, vulnerability, and niche overlap) and simulated network indices of web‐building spiders.
**Table S5:** The comparison between observed network indices (generality, vulnerability, and niche overlap) and simulated network indices of hunting spiders.
**Table S6:** Forty pairs of primer for amplification of spiders and their prey.
**Figure S1:** Relationship among predictors in linear models.

## Data Availability

Data available from the Science Data Bank https://doi.org/10.57760/sciencedb.26608 (Zhu, 2025) and BEF‐China project database https://data.botanik.uni‐halle.de/bef‐china/datasets/688.
