# Supplementary material for: Bottom‐up and top‐down effects combine to drive predator–prey interactions in a forest biodiversity experiment
Source: J Anim Ecol. 2025 Jul 7;94(10):2035–46. doi: 10.1111/1365-2656.70103 (PMC12484389; doi:10.1111/1365-2656.70103)
Supplement: Supplementary file 1 — Table S1: The functional traits, measurement and ecological functions of spiders. Table S2: The functional traits and ecological functions of leaf traits. Table S3: The comparison between observed network indices (generality, vulnerability, and niche overlap) and simulated network indices of all spiders. Table S4: The comparison between observed network indices (generality, vulnerability, and niche overlap) and simulated network indices of web‐building spiders. Table S5: The comparison between observed network indices (generality, vulnerability, and niche overlap) and simulated network indices of hunting spiders. Table S6: Forty pairs of primer for amplification of spiders and their prey. Figure S1: Relationship among predictors in linear models. [file JANE-94-2035-s001.docx]

**Bottom-up and top-down effects combine to drive predator-prey interactions in a forest biodiversity experiment**

**Supplementary Information**

**Chen et al.**

**MATERIALS AND METHODS**

**Study site**

This site represents a typical subtropical forest, with a mean annual temperature of 16.7 ℃ and an average annual precipitation of around 1880 mm (Yang *et al.* 2013). The “BEF-China” experiment covers 50 ha of designed and planted forest with 40 evergreen broad-leaved tree species and two economic tree species. The experiment comprises two sites (site A and site B), each containing a tree species pool of 24 species, with 8 species overlapping between these two sites. The two sites consist of 566 plots along a tree species diversity gradient including plots with 1, 2, 4, 8, 16, or 24 tree species. Each plot is 25.8×25.8 m in size, with 400 trees planted in a grid of 20×20 trees with 1.29 m distance between trees (see Bruelheide *et al.* 2014).

**Plant diversity**

Morphological leaf traits included specific leaf area (SLA), leaf dry matter content (LDMC), leaf toughness (LT), leaf area (LA), and chemical leaf traits included leaf carbon (C) concentrations, the ratio of leaf carbon to nitrogen (C:N) concentrations, leaf potassium (K) content, leaf calcium (Ca) content, leaf sodium (Na) content and leaf phosphorus (P) content. The potential ecological functions of each leaf traits were listed in Table S2. All traits were measured adhering to standard protocols (Pérez-Harguindeguy *et al.* 2013). Leaf traits were measured on at least five individuals of sun-exposed leaves per tree species, which were conducted by Kröber, Heklau and Bruelheide (2015). The plant VD was calculated as the mean pairwise dissimilarity of tree height and crown projection area among all individuals at the center (6×6 trees per plot in monocultures and 2 species mixtures, or 12×12 trees per plot in mixtures with more tree species) per plot calculated based on Rao's Q (Schuldt *et al.* 2019).

**REFERENCES**

Birkhofer, K., Djoudi, E.A., Schnerch, B. & Michalko, R. (2022) Climatic conditions and functional traits affect spider diets in agricultural and non-agricultural habitats worldwide. *Ecography,* **2022,** e06090.

Blumenthal, D.M., Mueller, K.E., Kray, J.A., Ocheltree, T.W., Augustine, D.J. & Wilcox, K.R. (2020) Traits link drought resistance with herbivore defence and plant economics in semi-arid grasslands: The central roles of phenology and leaf dry matter content. *Journal of Ecology,* **108,** 2336-2351.

Borer, E.T., Lind, E.M., Firn, J., Seabloom, E.W., Anderson, T.M., Bakker, E.S., Biederman, L., La Pierre, K.J., MacDougall, A.S., Moore, J.L., Risch, A.C., Schutz, M. & Stevens, C.J. (2019) More salt, please: global patterns, responses and impacts of foliar sodium in grasslands. *Ecology Letters,* **22,** 1136-1144.

Brousseau, P.-M., Gravel, D. & Handa, I.T. (2019) Traits of litter-dwelling forest arthropod predators and detritivores covary spatially with traits of their resources. *Ecology,* **100,** e02815.

Bruelheide, H., Nadrowski, K., Assmann, T., Bauhus, J., Both, S., Buscot, F., Chen, X.Y., Ding, B.Y., Durka, W., Erfmeier, A., Gutknecht, J.L.M., Guo, D.L., Guo, L.D., Haerdtle, W., He, J.S., Klein, A.M., Kuehn, P., Liang, Y., Liu, X.J., Michalski, S., Niklaus, P.A., Pei, K.Q., Scherer Lorenzen, M., Scholten, T., Schuldt, A., Seidler, G., Trogisch, S., von Oheimb, G., Welk, E., Wirth, C., Wubet, T., Yang, X.F., Yu, M.J., Zhang, S.R., Zhou, H.Z., Fischer, M., Ma, K.P. & Schmid, B. (2014) Designing forest biodiversity experiments: general considerations illustrated by a new large experiment in subtropical China. *Methods in Ecology and Evolution,* **5,** 74-89.

Ferreira-Sousa, L., Rocha, P.N., Motta, P.C. & Gawryszewski, F.M. (2021) Shaped by the Sun: the effect of exposure to sunlight on the evolution of spider bodies. *Biology Letters,* **17,** 20210369.

Gonçalves-Souza, T., Diniz-Filho, J.A.F. & Romero, G.Q. (2014) Disentangling the Phylogenetic and Ecological Components of Spider Phenotypic Variation. *PLoS One,* **9,** e89314.

Gu, H., Wang, H., Liu, M., Shangguan, Z., Shi, H., Xu, W., Ren, F., Zhu, J. & He, J.-S. (2022) Leaf N:P stoichiometry overrides the effect of individual nutrient content on insect herbivore population dynamics in a Tibetan alpine grassland. *Agriculture, Ecosystems & Environment,* **336,** 108032.

Jenkins, D.G., Brescacin, C.R., Duxbury, C.V., Elliott, J.A., Evans, J.A., Grablow, K.R., Hillegass, M., Lyon, B.N., Metzger, G.A., Olandese, M.L., Pepe, D., Silvers, G.A., Suresch, H.N., Thompson, T.N., Trexler, C.M., Williams, G.E., Williams, N.C. & Williams, S.E. (2007) Does size matter for dispersal distance? *Global Ecology and Biogeography,* **16,** 415-425.

Jetz, W., Carbone, C., Fulford, J. & Brown, J.H. (2004) The scaling of animal space use. *Science,* **306,** 266-268.

Kröber, W., Heklau, H. & Bruelheide, H. (2015) Leaf morphology of 40 evergreen and deciduous broadleaved subtropical tree species and relationships to functional ecophysiological traits. *Plant Biology,* **17,** 373-383.

Kumar, A., Panwar, R., Singh, A. & Singh, I.K. (2020) Role of Calcium Signalling During Plant–Herbivore Interaction. *Plant Stress Biology: Strategies and Trends* (eds B. Giri & M.P. Sharma), pp. 491-510. Springer Singapore, Singapore.

O'Neil, R.J. & Wiedenmann, R.N. (1987) Adaptations of Arthropod Predators to Agricultural Systems. *The Florida Entomologist,* **70,** 40-48.

Pérez-Harguindeguy, N., Díaz, S., Garnier, E., Lavorel, S., Poorter, H., Jaureguiberry, P., Bret-Harte, M.S., Cornwell, W.K., Craine, J.M., Gurvich, D.E., Urcelay, C., Veneklaas, E.J., Reich, P.B., Poorter, L., Wright, I.J., Ray, P., Enrico, L., Pausas, J.G., de Vos, A.C., Buchmann, N., Funes, G., Quétier, F., Hodgson, J.G., Thompson, K., Morgan, H.D., ter Steege, H., Sack, L., Blonder, B., Poschlod, P., Vaieretti, M.V., Conti, G., Staver, A.C., Aquino, S. & Cornelissen, J.H.C. (2013) New handbook for standardised measurement of plant functional traits worldwide. *Australian Journal of Botany,* **61**.

Pérez-Harguindeguy, N., Díaz, S., Vendramini, F., Cornelissen, J.H.C., Gurvich, D.E. & Cabido, M. (2003) Leaf traits and herbivore selection in the field and in cafeteria experiments. *Austral Ecology,* **28,** 642-650.

Poorter, L., van de Plassche, M., Willems, S. & Boot, R.G. (2004) Leaf traits and herbivory rates of tropical tree species differing in successional status. *Plant Biol (Stuttg),* **6,** 746-754.

Santonja, M., Aupic-Samain, A., Forey, E. & Chauvat, M. (2018) Increasing temperature and decreasing specific leaf area amplify centipede predation impact on Collembola. *European Journal of Soil Biology,* **89,** 9-13.

Schmitz, O.J. (2003) Top predator control of plant biodiversity and productivity in an old-field ecosystem. *Ecology Letters,* **6,** 156-163.

Schmitz, O.J. (2009) Effects of predator functional diversity on grassland ecosystem function. *Ecology,* **90,** 2339-2345.

Schuldt, A., Ebeling, A., Kunz, M., Staab, M., Guimaraes-Steinicke, C., Bachmann, D., Buchmann, N., Durka, W., Fichtner, A., Fornoff, F., Haerdtle, W., Hertzog, L.R., Klein, A.-M., Roscher, C., Schaller, J., von Oheimb, G., Weigelt, A., Weisser, W., Wirth, C., Zhang, J.Y., Bruelheide, H. & Eisenhauer, N. (2019) Multiple plant diversity components drive consumer communities across ecosystems. *Nature Communications,* **10,** 1460.

Urban, M.C. (2007) Predator size and phenology shape prey survival in temporary ponds. *Oecologia,* **154,** 571-580.

Wang, Y. & Wu, W.-H. (2017) Regulation of potassium transport and signaling in plants. *Current opinion in plant biology,* **39,** 123-128.

Yang, X.F., Bauhus, J., Both, S., Fang, T., Haerdtle, W., Kroeber, W., Ma, K.P., Nadrowski, K., Pei, K.Q., Scherer-Lorenzen, M., Scholten, T., Seidler, G., Schmid, B., von Oheimb, G. & Bruelheide, H. (2013) Establishment success in a forest biodiversity and ecosystem functioning experiment in subtropical China (BEF-China). *European Journal of Forest Research,* **132,** 593-606.


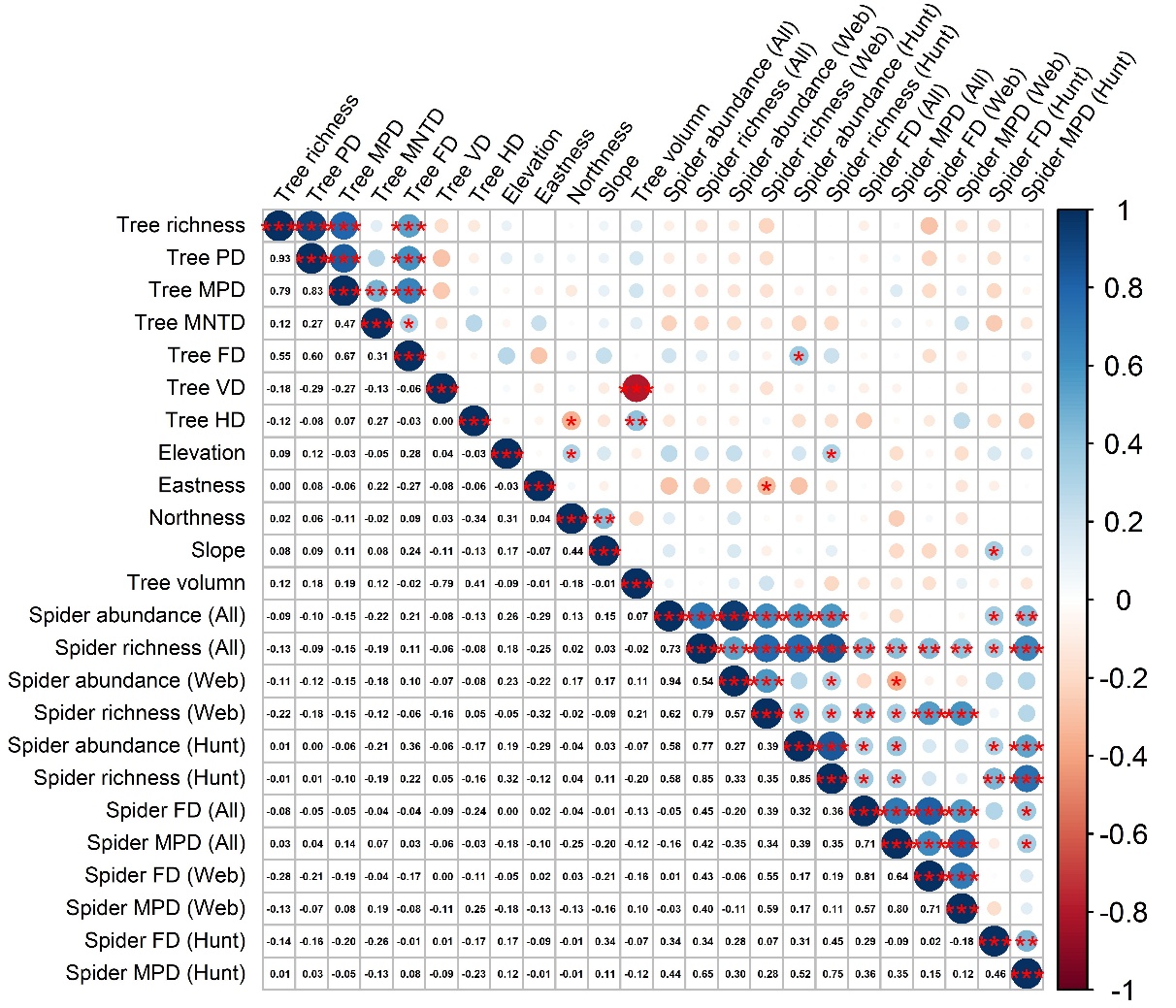


**Figure S1** Relationship among predictors in linear models. Values are Pearson correlation coefficient *r*. Significance is denoted by asterisks (*** P < 0.001, ** P < 0.01, * P < 0.05).

**Table S1** The functional traits, measurement and ecological functions of spiders.

| **Traits** | **Measurement** | **Ecological functions** | **Reference** |
| --- | --- | --- | --- |
| **Body shape** | Body length divided by width of carapace | Related to locomotion, dispersal, and thermoregulation. | Jenkins et al. (2007); Ferreira-Sousa et al. (2021) |
| **Flatness** | Height of carapace divided by body length | Related to locomotion, hiding and predation. | Gonçalves-Souza, Diniz-Filho and Romero (2014) |
| **Biomass** | Mean dry weight | Related to space use and resource utilization. | Jetz et al. (2004); Birkhofer et al. (2022) |
| **Hunting mode** | Web-building or hunting | Reflecting different interaction pathways between spiders and other trophic levels. | Schmitz (2003); Schmitz (2009) |
| **Phenology** | Three levels (1, 2, 3) based on the occurrence of spider at three seasons (April, June and September) | Duration that spiders exert hunting pressure on their prey. | Urban (2007) |

**Table S2** The functional traits and ecological functions of leaf traits.

| **Traits** | **Ecological functions** | **Reference** |
| --- | --- | --- |
| **Specific leaf area (SLA)** | Reflects less spatial refuges and higher probability to encounter predators and often positively related to herbivory. | Pérez-Harguindeguy et al. (2003); Santonja et al. (2018) |
| **Leaf dry matter content (LDMC)** | High leaf dry matter content reduces herbivory, while providing stronger physical structures for predators (e.g. for web attachment). | Blumenthal et al. (2020) |
| **Leaf toughness (LT)** | Reflects palatability of leaf, which could influence predators through impacting herbivores and decomposers. | Brousseau, Gravel and Handa (2019) |
| **Leaf area (LA)** | Defines the searching area of predators and may influence web attachment of different types of web builders. | O'Neil and Wiedenmann (1987) |
| **Leaf carbon (C) concentrations** | Related to palatability and may therefore strongly influence the availability and community composition of prey organisms (both herbivores and decomposers). | Poorter et al. (2004) |
| **Ratio of leaf carbon to nitrogen (C:N) concentrations** | Related to leaf palatability. | Pérez-Harguindeguy et al. (2003) |
| **Leaf potassium (K) content** | Related to leaf palatability by influencing the formation of soluble sugars, organic acids, amino acids, and amides of plant. | Wang and Wu (2017) |
| **Leaf calcium (Ca) content** | Related to leaf defense and further influence herbivores. | Kumar et al. (2020) |
| **Leaf sodium (Na) content** | Essential element for herbivores, could influence foraging preference. | Borer et al. (2019) |
| **Leaf phosphorus (P) content** | Phosphorus promotes plant growth and further influence diversity and composition of prey communities. | Gu et al. (2022) |

**Table S3** The comparison between observed network indices (generality, vulnerability, and niche overlap) and simulated network indices of all spiders. A p-value lower than 0.05 indicates a significant difference between observed and simulated values, which is highlighted in bold.

| Plot | Generality (0.77) | | |  | Vulnerability (0.95) | | |  | Niche overlap (1) | | |
| --- | --- | --- | --- | --- | --- | --- | --- | --- | --- | --- | --- |
|  | Observe | Null (mean) | *P* |  | Observe | Null (mean) | *P* |  | Observe | Null (mean) | *P* |
| A_E34 | 8.51 | 3.73 | **0.000** |  | 1.08 | 3.72 | **0.000** |  | 0.00 | 3.73 | **0.000** |
| A_E33 | 4.43 | 3.52 | **0.001** |  | 1.48 | 3.52 | **0.000** |  | 0.02 | 3.52 | **0.000** |
| A_C32 | 9.62 | 4.05 | **0.000** |  | 1.00 | 4.05 | **0.000** |  | 0.00 | 4.05 | **0.000** |
| A_E31 | 3.00 | 3.00 | **0.000** |  | 1.00 | 3.00 | **0.000** |  | 0.00 | 3.00 | **0.000** |
| A_H31 | 14.63 | 7.84 | **0.000** |  | 1.00 | 7.84 | **0.000** |  | 0.00 | 7.84 | **0.000** |
| A_F27 | 3.20 | 2.46 | **0.008** |  | 1.55 | 2.46 | **0.000** |  | 0.08 | 2.46 | **0.000** |
| A_I27 | 3.14 | 3.14 | **0.000** |  | 1.29 | 3.14 | **0.000** |  | 0.05 | 3.14 | **0.000** |
| A_I28 | 4.20 | 2.98 | **0.001** |  | 2.00 | 2.98 | **0.000** |  | 0.14 | 2.98 | **0.000** |
| A_G24 | 1.67 | 1.67 | **0.000** |  | 1.33 | 1.67 | **0.000** |  | 0.08 | 1.67 | **0.000** |
| A_L11 | 3.88 | 3.06 | **0.002** |  | 2.08 | 3.06 | **0.000** |  | 0.07 | 3.06 | **0.000** |
| A_F21 | 2.94 | 2.41 | **0.011** |  | 2.08 | 2.41 | **0.002** |  | 0.09 | 2.41 | **0.000** |
| A_N13 | 5.48 | 3.78 | **0.000** |  | 1.27 | 3.78 | **0.000** |  | 0.03 | 3.78 | **0.000** |
| A_N11 | 5.56 | 4.13 | **0.010** |  | 1.18 | 4.14 | **0.000** |  | 0.07 | 4.14 | **0.000** |
| A_N9 | 2.88 | 2.88 | **0.000** |  | 1.75 | 2.88 | **0.000** |  | 0.17 | 2.88 | **0.000** |
| A_N8 | 2.64 | 2.59 | 0.357 |  | 1.21 | 2.60 | **0.000** |  | 0.03 | 2.59 | **0.000** |
| A_W14 | 1.29 | 1.29 | **0.000** |  | 1.00 | 1.29 | **0.000** |  | 0.00 | 1.29 | **0.000** |
| A_X/W12 | 3.81 | 3.36 | **0.048** |  | 1.67 | 3.36 | **0.000** |  | 0.06 | 3.36 | **0.000** |
| A_S10 | 3.00 | 3.00 | **0.000** |  | 1.46 | 3.00 | **0.000** |  | 0.10 | 3.00 | **0.000** |
| A_T15 | 3.91 | 3.10 | **0.004** |  | 1.98 | 3.10 | **0.000** |  | 0.07 | 3.10 | **0.000** |
| A_R14 | 1.73 | 1.72 | 0.328 |  | 1.89 | 1.72 | 0.063 |  | 0.06 | 1.72 | **0.000** |
| A_Q21 | 2.44 | 2.16 | 0.055 |  | 2.29 | 2.16 | 0.193 |  | 0.13 | 2.16 | **0.000** |
| A_K19 | 3.89 | 3.89 | **0.000** |  | 1.00 | 3.89 | **0.000** |  | 0.00 | 3.89 | **0.000** |
| A_J21 | 3.67 | 3.56 | 0.284 |  | 1.92 | 3.56 | **0.000** |  | 0.09 | 3.56 | **0.000** |
| A_L22 | 9.95 | 4.07 | **0.000** |  | 1.73 | 4.06 | **0.000** |  | 0.14 | 4.06 | **0.000** |
| A_O22 | 4.54 | 3.88 | 0.054 |  | 1.55 | 3.89 | **0.000** |  | 0.20 | 3.88 | **0.000** |
| A_P26 | 8.94 | 4.46 | **0.000** |  | 1.72 | 4.46 | **0.000** |  | 0.09 | 4.46 | **0.000** |
| B_G28 | 4.24 | 4.24 | **0.000** |  | 1.29 | 4.24 | **0.000** |  | 0.05 | 4.24 | **0.000** |
| B_J29 | 3.00 | 3.00 | **0.000** |  | 1.00 | 3.00 | **0.000** |  | 0.00 | 3.00 | **0.000** |
| B_I25 | 4.97 | 3.73 | **0.003** |  | 1.52 | 3.73 | **0.000** |  | 0.07 | 3.73 | **0.000** |
| B_M29 | 2.37 | 2.70 | 0.172 |  | 1.22 | 2.70 | **0.000** |  | 0.06 | 2.70 | **0.000** |
| B_M22 | 3.18 | 3.18 | **0.000** |  | 1.00 | 3.18 | **0.000** |  | 0.00 | 3.18 | **0.000** |
| B_M24 | 2.00 | 2.00 | **0.000** |  | 1.00 | 2.00 | **0.000** |  | 0.00 | 2.00 | **0.000** |
| B_O31 | 9.17 | 9.17 | **0.000** |  | 1.00 | 9.17 | **0.000** |  | 0.00 | 9.17 | **0.000** |
| B_O27 | 3.42 | 2.89 | 0.055 |  | 1.00 | 2.89 | **0.000** |  | 0.00 | 2.89 | **0.000** |
| B_Q17 | 2.24 | 1.97 | 0.084 |  | 1.46 | 1.97 | **0.000** |  | 0.10 | 1.97 | **0.000** |
| B_S22 | 4.19 | 3.30 | **0.010** |  | 1.18 | 3.31 | **0.000** |  | 0.02 | 3.31 | **0.000** |
| B_R29 | 2.33 | 2.33 | **0.000** |  | 1.40 | 2.33 | **0.000** |  | 0.05 | 2.33 | **0.000** |
| B_W11 | 3.58 | 3.58 | 0.374 |  | 1.38 | 3.57 | **0.000** |  | 0.11 | 3.57 | **0.000** |
| B_U16 | 3.25 | 3.25 | **0.000** |  | 1.00 | 3.25 | **0.000** |  | 0.00 | 3.25 | **0.000** |
| B_V19 | 2.43 | 2.43 | **0.000** |  | 1.00 | 2.43 | **0.000** |  | 0.00 | 2.43 | **0.000** |
| B_V23 | 3.62 | 3.62 | **0.000** |  | 1.15 | 3.62 | **0.000** |  | 0.04 | 3.62 | **0.000** |
| B_N5 | 9.81 | 5.36 | **0.000** |  | 1.29 | 5.36 | **0.000** |  | 0.13 | 5.36 | **0.000** |
| B_M7 | 5.18 | 4.51 | 0.050 |  | 1.48 | 4.53 | **0.000** |  | 0.09 | 4.52 | **0.000** |
| B_R3 | 4.02 | 2.51 | **0.000** |  | 1.72 | 2.51 | **0.000** |  | 0.17 | 2.51 | **0.000** |

**Table S4** The comparison between observed network indices (generality, vulnerability, and niche overlap) and simulated network indices of web-building spiders. A p-value lower than 0.05 indicates a significant difference between observed and simulated values, which is highlighted in bold.

| Plot | Generality (0.67) | | |  | Vulnerability (1) | | |  | Niche overlap (1) | | |
| --- | --- | --- | --- | --- | --- | --- | --- | --- | --- | --- | --- |
|  | Observe | Null (mean) | *P* |  | Observe | Null (mean) | *P* |  | Observe | Null (mean) | *P* |
| A_E34 | 2.62 | 2.62 | **0.000** |  | 1.00 | 2.62 | **0.000** |  | 0.00 | 2.62 | **0.000** |
| A_E33 | 4.52 | 3.67 | **0.011** |  | 1.14 | 3.67 | **0.000** |  | 0.01 | 3.67 | **0.000** |
| A_F27 | 3.76 | 2.69 | **0.021** |  | 1.30 | 2.70 | **0.000** |  | 0.20 | 2.70 | **0.000** |
| A_I27 | 1.50 | 1.50 | **0.000** |  | 1.00 | 1.50 | **0.000** |  | 0.00 | 1.50 | **0.000** |
| A_I28 | 2.66 | 2.64 | 0.330 |  | 1.00 | 2.64 | **0.000** |  | 0.00 | 2.64 | **0.000** |
| A_L11 | 4.25 | 3.10 | **0.003** |  | 1.48 | 3.10 | **0.000** |  | 0.08 | 3.10 | **0.000** |
| A_F21 | 2.86 | 2.73 | 0.219 |  | 1.93 | 2.73 | **0.000** |  | 0.19 | 2.73 | **0.000** |
| A_N13 | 4.20 | 2.89 | **0.002** |  | 1.00 | 2.89 | **0.000** |  | 0.00 | 2.89 | **0.000** |
| A_W14 | 1.00 | 1.00 | **0.000** |  | 1.00 | 1.00 | **0.000** |  | 0.00 | 1.00 | **0.000** |
| A_X/W12 | 2.88 | 2.72 | 0.245 |  | 1.08 | 2.72 | **0.000** |  | 0.00 | 2.72 | **0.000** |
| A_S10 | 2.71 | 2.71 | **0.000** |  | 1.00 | 2.71 | **0.000** |  | 0.00 | 2.71 | **0.000** |
| A_T15 | 4.38 | 3.08 | **0.001** |  | 1.61 | 3.08 | **0.000** |  | 0.08 | 3.08 | **0.000** |
| A_R14 | 1.89 | 1.85 | 0.240 |  | 1.39 | 1.85 | **0.000** |  | 0.05 | 1.84 | **0.000** |
| A_Q21 | 2.14 | 2.14 | **0.000** |  | 1.86 | 2.14 | **0.000** |  | 0.33 | 2.14 | **0.000** |
| A_J21 | 3.66 | 3.86 | 0.335 |  | 2.06 | 3.86 | **0.000** |  | 0.42 | 3.87 | **0.000** |
| A_L22 | 2.45 | 2.51 | 0.486 |  | 1.48 | 2.52 | **0.000** |  | 0.22 | 2.51 | **0.000** |
| A_P26 | 2.95 | 2.78 | 0.285 |  | 1.28 | 2.79 | **0.000** |  | 0.12 | 2.78 | **0.000** |
| B_G28 | 3.40 | 3.40 | **0.000** |  | 1.00 | 3.40 | **0.000** |  | 0.00 | 3.40 | **0.000** |
| B_I25 | 5.18 | 3.81 | **0.007** |  | 1.41 | 3.81 | **0.000** |  | 0.13 | 3.81 | **0.000** |
| B_M29 | 2.52 | 2.52 | 0.372 |  | 1.00 | 2.51 | **0.000** |  | 0.00 | 2.51 | **0.000** |
| B_M22 | 2.71 | 2.71 | **0.000** |  | 1.00 | 2.71 | **0.000** |  | 0.00 | 2.71 | **0.000** |
| B_O31 | 1.00 | 1.00 | **0.000** |  | 1.00 | 1.00 | **0.000** |  | 0.00 | 1.00 | **0.000** |
| B_O27 | 3.66 | 3.35 | 0.159 |  | 1.00 | 3.35 | **0.000** |  | 0.00 | 3.35 | **0.000** |
| B_S22 | 3.42 | 2.88 | **0.050** |  | 1.00 | 2.89 | **0.000** |  | 0.00 | 2.89 | **0.000** |
| B_R29 | 2.20 | 2.20 | **0.000** |  | 1.00 | 2.20 | **0.000** |  | 0.00 | 2.20 | **0.000** |
| B_U16 | 3.25 | 3.25 | **0.000** |  | 1.00 | 3.25 | **0.000** |  | 0.00 | 3.25 | **0.000** |
| B_V23 | 3.80 | 3.80 | **0.000** |  | 1.00 | 3.80 | **0.000** |  | 0.00 | 3.80 | **0.000** |

**Table S5** The comparison between observed network indices (generality, vulnerability, and niche overlap) and simulated network indices of hunting spiders. A p-value lower than 0.05 indicates a significant difference between observed and simulated values, which is highlighted in bold.

| Plot | Generality (0.8) | | |  | Vulnerability (1) | | |  | Niche overlap (1) | | |
| --- | --- | --- | --- | --- | --- | --- | --- | --- | --- | --- | --- |
|  | Observe | Null (mean) | *P* |  | Observe | Null (mean) | *P* |  | Observe | Null (mean) | *P* |
| A_E34 | 12.05 | 7.22 | **0.000** |  | 1.00 | 7.22 | **0.000** |  | 0.00 | 7.22 | **0.000** |
| A_E33 | 4.35 | 3.31 | **0.003** |  | 1.31 | 3.31 | **0.000** |  | 0.03 | 3.31 | **0.000** |
| A_C32 | 10.62 | 5.01 | **0.000** |  | 1.00 | 5.01 | **0.000** |  | 0.00 | 5.01 | **0.000** |
| A_F27 | 2.50 | 2.50 | **0.000** |  | 1.00 | 2.50 | **0.000** |  | 0.00 | 2.50 | **0.000** |
| A_I27 | 3.80 | 3.80 | **0.000** |  | 1.40 | 3.80 | **0.000** |  | 0.23 | 3.80 | **0.000** |
| A_I28 | 4.88 | 3.07 | **0.000** |  | 1.69 | 3.08 | **0.000** |  | 0.20 | 3.07 | **0.000** |
| A_G24 | 1.50 | 1.50 | **0.000** |  | 1.00 | 1.50 | **0.000** |  | 0.00 | 1.50 | **0.000** |
| A_L11 | 3.51 | 2.94 | **0.029** |  | 1.54 | 2.94 | **0.000** |  | 0.06 | 2.94 | **0.000** |
| A_F21 | 3.05 | 2.21 | **0.006** |  | 1.18 | 2.20 | **0.000** |  | 0.07 | 2.20 | **0.000** |
| A_N13 | 6.03 | 4.25 | **0.001** |  | 1.19 | 4.24 | **0.000** |  | 0.04 | 4.24 | **0.000** |
| A_N11 | 6.20 | 4.20 | **0.002** |  | 1.22 | 4.20 | **0.000** |  | 0.13 | 4.21 | **0.000** |
| A_N9 | 2.50 | 2.50 | **0.000** |  | 2.00 | 2.50 | **0.000** |  | 0.23 | 2.50 | **0.000** |
| A_N8 | 2.71 | 2.71 | **0.000** |  | 1.00 | 2.71 | **0.000** |  | 0.00 | 2.71 | **0.000** |
| A_W14 | 1.50 | 1.50 | **0.000** |  | 1.00 | 1.50 | **0.000** |  | 0.00 | 1.50 | **0.000** |
| A_X/W12 | 5.06 | 5.13 | 0.429 |  | 1.58 | 5.12 | **0.000** |  | 0.44 | 5.13 | **0.000** |
| A_T15 | 3.18 | 3.19 | 0.454 |  | 1.18 | 3.20 | **0.000** |  | 0.03 | 3.19 | **0.000** |
| A_R14 | 1.52 | 1.80 | 0.228 |  | 1.30 | 1.80 | **0.000** |  | 0.04 | 1.79 | **0.000** |
| A_Q21 | 2.61 | 2.16 | **0.026** |  | 1.39 | 2.15 | **0.000** |  | 0.05 | 2.16 | **0.000** |
| A_J21 | 3.67 | 3.44 | 0.208 |  | 1.12 | 3.44 | **0.000** |  | 0.01 | 3.44 | **0.000** |
| A_L22 | 12.45 | 5.36 | **0.000** |  | 1.13 | 5.37 | **0.000** |  | 0.04 | 5.37 | **0.000** |
| A_P26 | 10.74 | 5.31 | **0.000** |  | 1.20 | 5.30 | **0.000** |  | 0.04 | 5.30 | **0.000** |
| B_G28 | 5.00 | 5.00 | **0.000** |  | 1.00 | 5.00 | **0.000** |  | 0.00 | 5.00 | **0.000** |
| B_I25 | 4.56 | 4.56 | **0.000** |  | 1.00 | 4.56 | **0.000** |  | 0.00 | 4.56 | **0.000** |
| B_M24 | 2.20 | 2.20 | **0.000** |  | 1.00 | 2.20 | **0.000** |  | 0.00 | 2.20 | **0.000** |
| B_O31 | 10.33 | 10.33 | **0.000** |  | 1.00 | 10.33 | **0.000** |  | 0.00 | 10.33 | **0.000** |
| B_Q17 | 2.34 | 2.13 | 0.143 |  | 1.50 | 2.13 | **0.000** |  | 0.16 | 2.13 | **0.000** |
| B_S22 | 4.67 | 3.26 | **0.003** |  | 1.00 | 3.26 | **0.000** |  | 0.00 | 3.26 | **0.000** |
| B_R29 | 2.40 | 2.40 | **0.000** |  | 1.60 | 2.40 | **0.000** |  | 0.13 | 2.40 | **0.000** |
| B_M7 | 4.79 | 4.45 | 0.155 |  | 1.69 | 4.45 | **0.000** |  | 0.22 | 4.44 | **0.000** |
| B_R3 | 4.27 | 2.77 | **0.000** |  | 1.78 | 2.77 | **0.000** |  | 0.28 | 2.77 | **0.000** |

**Table S6** Forty pairs of primer for amplification of spiders and their prey. Capital letters represent sequences of primer ZBJ-ArtF1c and ZBJ-ArtR2c. The lowercase letters represent the corresponding barcode.

| Primer name | Forward primer | Reverse primer |
| --- | --- | --- |
| Primer_01 | gtcacgtcAGATATTGGAACWTTATATTTTATTTTTGG | GGAGGATTTGGWAATTGATTAGTWtagcatag |
| Primer_02 | gactgatgAGATATTGGAACWTTATATTTTATTTTTGG | GGAGGATTTGGWAATTGATTAGTWcagcgcga |
| Primer_03 | agactatgAGATATTGGAACWTTATATTTTATTTTTGG | GGAGGATTTGGWAATTGATTAGTWactgtgct |
| Primer_04 | gcgtcagcAGATATTGGAACWTTATATTTTATTTTTGG | GGAGGATTTGGWAATTGATTAGTWactagcta |
| Primer_05 | tgacatcaAGATATTGGAACWTTATATTTTATTTTTGG | GGAGGATTTGGWAATTGATTAGTWgtagcact |
| Primer_06 | acatgtgtAGATATTGGAACWTTATATTTTATTTTTGG | GGAGGATTTGGWAATTGATTAGTWtgtatacg |
| Primer_07 | cgtatacaAGATATTGGAACWTTATATTTTATTTTTGG | GGAGGATTTGGWAATTGATTAGTWacgactcg |
| Primer_08 | atgatcgcAGATATTGGAACWTTATATTTTATTTTTGG | GGAGGATTTGGWAATTGATTAGTWatcatgtg |
| Primer_09 | aaaggtccAGATATTGGAACWTTATATTTTATTTTTGG | GGAGGATTTGGWAATTGATTAGTWaaaggtcc |
| Primer_10 | aacgacacAGATATTGGAACWTTATATTTTATTTTTGG | GGAGGATTTGGWAATTGATTAGTWaacgacac |
| Primer_11 | aagacctgAGATATTGGAACWTTATATTTTATTTTTGG | GGAGGATTTGGWAATTGATTAGTWaagacctg |
| Primer_12 | aagcatcgAGATATTGGAACWTTATATTTTATTTTTGG | GGAGGATTTGGWAATTGATTAGTWaagcatcg |
| Primer_13 | aatagggcAGATATTGGAACWTTATATTTTATTTTTGG | GGAGGATTTGGWAATTGATTAGTWaatagggc |
| Primer_14 | acacactgAGATATTGGAACWTTATATTTTATTTTTGG | GGAGGATTTGGWAATTGATTAGTWacacactg |
| Primer_15 | acgctactAGATATTGGAACWTTATATTTTATTTTTGG | GGAGGATTTGGWAATTGATTAGTWacgctact |
| Primer_16 | acgttgtcAGATATTGGAACWTTATATTTTATTTTTGG | GGAGGATTTGGWAATTGATTAGTWacgttgtc |
| Primer_17 | actttcggAGATATTGGAACWTTATATTTTATTTTTGG | GGAGGATTTGGWAATTGATTAGTWactttcgg |
| Primer_18 | agactgagAGATATTGGAACWTTATATTTTATTTTTGG | GGAGGATTTGGWAATTGATTAGTWagactgag |
| Primer_19 | agcaggaaAGATATTGGAACWTTATATTTTATTTTTGG | GGAGGATTTGGWAATTGATTAGTWagcaggaa |
| Primer_20 | agcgtctaAGATATTGGAACWTTATATTTTATTTTTGG | GGAGGATTTGGWAATTGATTAGTWagcgtcta |
| Primer_21 | aggggtatAGATATTGGAACWTTATATTTTATTTTTGG | GGAGGATTTGGWAATTGATTAGTWaggggtat |
| Primer_22 | aggtgacaAGATATTGGAACWTTATATTTTATTTTTGG | GGAGGATTTGGWAATTGATTAGTWaggtgaca |
| Primer_23 | agtcttgcAGATATTGGAACWTTATATTTTATTTTTGG | GGAGGATTTGGWAATTGATTAGTWagtcttgc |
| Primer_24 | agtgcagtAGATATTGGAACWTTATATTTTATTTTTGG | GGAGGATTTGGWAATTGATTAGTWagtgcagt |
| Primer_25 | atacgggaAGATATTGGAACWTTATATTTTATTTTTGG | GGAGGATTTGGWAATTGATTAGTWatacggga |
| Primer_26 | atagtccgAGATATTGGAACWTTATATTTTATTTTTGG | GGAGGATTTGGWAATTGATTAGTWatagtccg |
| Primer_27 | atatcgccAGATATTGGAACWTTATATTTTATTTTTGG | GGAGGATTTGGWAATTGATTAGTWatatcgcc |
| Primer_28 | atcagcgtAGATATTGGAACWTTATATTTTATTTTTGG | GGAGGATTTGGWAATTGATTAGTWatcagcgt |
| Primer_29 | atcccatgAGATATTGGAACWTTATATTTTATTTTTGG | GGAGGATTTGGWAATTGATTAGTWatcccatg |
| Primer_30 | caagcacaAGATATTGGAACWTTATATTTTATTTTTGG | GGAGGATTTGGWAATTGATTAGTWcaagcaca |
| Primer_31 | cacaagtcAGATATTGGAACWTTATATTTTATTTTTGG | GGAGGATTTGGWAATTGATTAGTWcacaagtc |
| Primer_32 | cactgtgtAGATATTGGAACWTTATATTTTATTTTTGG | GGAGGATTTGGWAATTGATTAGTWcactgtgt |
| Primer_33 | cagagtagAGATATTGGAACWTTATATTTTATTTTTGG | GGAGGATTTGGWAATTGATTAGTWcagagtag |
| Primer_34 | catatcccAGATATTGGAACWTTATATTTTATTTTTGG | GGAGGATTTGGWAATTGATTAGTWcatatccc |
| Primer_35 | catcagcaAGATATTGGAACWTTATATTTTATTTTTGG | GGAGGATTTGGWAATTGATTAGTWcatcagca |
| Primer_36 | catgaaggAGATATTGGAACWTTATATTTTATTTTTGG | GGAGGATTTGGWAATTGATTAGTWcatgaagg |
| Primer_37 | ccacttcaAGATATTGGAACWTTATATTTTATTTTTGG | GGAGGATTTGGWAATTGATTAGTWccacttca |
| Primer_38 | ccatctacAGATATTGGAACWTTATATTTTATTTTTGG | GGAGGATTTGGWAATTGATTAGTWAccatcta |
| Primer_39 | ccatggtaAGATATTGGAACWTTATATTTTATTTTTGG | GGAGGATTTGGWAATTGATTAGTWccatggta |
| Primer_40 | ccgaaaacAGATATTGGAACWTTATATTTTATTTTTGG | GGAGGATTTGGWAATTGATTAGTWccgaaaac |
